# Methodological Challenges in Collecting Social and Behavioural Data Regarding the HIV Epidemic among Gay and Other Men Who Have Sex with Men in Australia

**DOI:** 10.1371/journal.pone.0113167

**Published:** 2014-11-19

**Authors:** Iryna B. Zablotska, Andrew Frankland, Martin Holt, John de Wit, Graham Brown, Bruce Maycock, Christopher Fairley, Garrett Prestage

**Affiliations:** 1 The Kirby Institute, The University of NSW Australia, Sydney, NSW, Australia; 2 School of Psychiatry, The University of NSW Australia, Sydney, NSW, Australia; 3 Centre for Social Research in Health, The University of NSW Australia, Sydney, NSW, Australia; 4 The Australian Research Centre in Sex, Health and Society, La Trobe University, Melbourne, VIC, Australia; 5 Curtin University, Perth, WA, Australia; 6 Central Clinical School, Monash University, Melbourne, VIC, Australia; 7 Melbourne Sexual Health Centre, Melbourne, VIC, Australia; Örebro University, Sweden

## Abstract

**Background:**

Behavioural surveillance and research among gay and other men who have sex with men (GMSM) commonly relies on non-random recruitment approaches. Methodological challenges limit their ability to accurately represent the population of adult GMSM. We compared the social and behavioural profiles of GMSM recruited via venue-based, online, and respondent-driven sampling (RDS) and discussed their utility for behavioural surveillance.

**Methods:**

Data from four studies were selected to reflect each recruitment method. We compared demographic characteristics and the prevalence of key indicators including sexual and HIV testing practices obtained from samples recruited through different methods, and population estimates from respondent-driven sampling partition analysis.

**Results:**

Overall, the socio-demographic profile of GMSM was similar across samples, with some differences observed in age and sexual identification. Men recruited through time-location sampling appeared more connected to the gay community, reported a greater number of sexual partners, but engaged in less unprotected anal intercourse with regular (UAIR) or casual partners (UAIC). The RDS sample overestimated the proportion of HIV-positive men and appeared to recruit men with an overall higher number of sexual partners. A single-website survey recruited a sample with characteristics which differed considerably from the population estimates with regards to age, ethnically diversity and behaviour. Data acquired through time-location sampling underestimated the rates of UAIR and UAIC, while RDS and online sampling both generated samples that underestimated UAIR. Simulated composite samples combining recruits from time-location and multi-website online sampling may produce characteristics more consistent with the population estimates, particularly with regards to sexual practices.

**Conclusion:**

Respondent-driven sampling produced the sample that was most consistent to population estimates, but this methodology is complex and logistically demanding. Time-location and online recruitment are more cost-effective and easier to implement; using these approaches in combination may offer the potential to recruit a more representative sample of GMSM.

## Introduction

In recent decades, a substantial body of research amongst gay men who have sex with men (GMSM) has accumulated, with a particular focus on mapping the trends in a range of social and behavioural factors. An emphasis on behavioural surveillance has been driven by the importance of monitoring behaviours relevant to the transmission of HIV and other sexually transmitted infections (STIs), providing data for targeted HIV and STI prevention policy, and for evaluating health-promotion efforts. [1] In countries with concentrated HIV epidemics among GMSM, there has been a strategic value in focusing on gay and bisexual men living in metropolitan areas [2], [3], given the concentration of new HIV infections within this group. [4] Also, accessing a broader, more representative sample of GMSM is challenging, and social-behavioural research and behavioural surveillance have been reliant on a limited number of recruitment methods such as convenience, time-location and online sampling. [5]–[8] A number of methodological issues persist in the field, including limited generalizability and a lack of certainty over how the samples generated by each recruitment approach differ from one another. [9], [10] The absence of a validated sampling frame limits the ability to evaluate these different recruitment approaches, and identify the methodology most likely to produce a representative sample. [11] These issues are critical, given that the utility of behavioural surveillance efforts rest on the identification of representative subgroups that allow researchers to gather reliable data. The importance of these data in guiding health policy and community-based education highlights the potential risk of incomplete or inaccurate surveillance data [12].

In recent years, peer-referral approaches such as Respondent-Driven Sampling (RDS) have become more widespread [13], offering both a methodology for accessing hard-to-reach populations as well as the potential to produce population estimates of key behavioural indicators. [14] Although not free from bias [15], these estimates provide novel social and behavioural data within the GMSM population, as well as an opportunity to explore differences in the behavioural profiles of GMSM accessed via different recruitment approaches. The current study aimed to assess and compare multiple recruitment strategies for studying social and behavioural factors relevant to HIV transmission among GMSM. Additionally, we sought to determine if each sample described the same population, to document the differences in the samples produced by each method, and to explore which method provided the optimal balance between reliability, representativeness, and cost.

## Methods

### Population and samples

The following data sources were selected to represent different recruitment methods:

1. *Gay Community Periodic Surveys (GCPS), funded by the State Departments of Health* in six Australian jurisdictions, are part of the national HIV behavioural surveillance system and have been used to collected data on HIV related behaviours among gay men annually since 1996. The methodology of data collection has been described previously. [16] Briefly, these repeated surveys employ convenience time-location sampling and recruit gay men at gay community venues, events and clinics. They collect information about socio-demographic characteristics of participants, their sexual partnerships and practices, illicit drug use and testing for HIV and STI. The core socio-demographic and behavioural questions have remained stable since the start of the surveys in 1996.
2. The study of Contemporary Norms in Networks and Communities of GMSM (CONNECT) was funded by the Australian National Health and Medical Research Council. This cross-sectional multi-site survey was specifically focused on: 1) investigating the patterns of connections between individuals in GMSM communities and assess how they shape HIV-related behaviours; 2) assessing the relationship between social norms and sexual practices, and 3) comparing the norms and patterns of behaviour in geographically and epidemiologically distinct GMSM populations in the capital cities of three Australian states New South Wales (NSW), Victoria and Western Australia, in order to identify local community norms and barriers to effective HIV prevention. This quantitative study recruited participants using two recruitment methods: RDS in stage I (CONNECT-I, February 2011- April 2012) and Internet-based recruitment in stage II (CONNECT-II, June - August 2012). The methods of CONNECT-I have been described previously. [17] The online recruitment for CONNECT-II was conducted using online advertisement and e-blast emails about the study to the membership of the Squirt website. This website offers its members the opportunity to meet other men for online connections and finding partners. The same data collection tool was used in both stages, and questions collecting information about socio-demographic characteristics, sexual practices and testing for HIV/STI were adopted from the GCPS. In this analysis, the sample recruited by CONNECT-I was used to examine the characteristics of an RDS sample.
3. As the source of sample(s) recruited online, we considered studies that satisfied the following criteria: 1) participants included men living in the same cities as the participants of the CONNECT-I and GCPS, 2) enrolment was conducted during approximately the same time-frame as in the latter two studies, 3) comparable data collection tools, particularly with respect to socio-demographic, behavioural and testing indicators, and 4) recruitment of GMSM online. The CONNECT-II sample was used as an example of a sample recruited specifically through a single website, and the Pleasure and Sexual Health (PASH) study provided a sample recruited through multiple websites. The PASH study was commissioned and funded by the Departments of Health in the states of NSW, Victoria, South Australia and Western Australia. Its design and methods have been described previously. [18] The study participants were recruited online, and quantitative socio-demographic and behavioural information was collected using tools developed by GCPS.

In all studies included in this analysis behavioural information was collected anonymously; clinical records were not used; personal identifying information was not collected, and participants were not asked to provide written informed consent. For each of the studies included in this analysis, approvals have been obtained from the appropriate Human Research Ethics Committees: for CONNECT – from the University of NSW Australia (HREC 09381) and Curtin University, Perth (SPH-04-2010)); for PASH – from the University of NSW Australia (HREC 07207), and for GCPS - from the University of NSW Australia (HREC 09209).

### Data analyses

We used the data from the selected datasets to compare the characteristics of the samples and the prevalence of sexual and testing practices among the participants. The variables of interest included the following socio-demographic factors: age in years (under 25 (reference group), 25–34, 35–44, 45–54 and 55 or more), ethnic background (Anglo-Australian versus Other), level of education (up to three years of high school (reference category), completed high school, tertiary diploma and university degree), having been tested for HIV in the past (Yes versus No), HIV serostatus (positive (reference group), negative or unknown/not sure). We also explored sexual identity of the participants (gay/bisexual (reference group), bisexual, heterosexual and other), indicators of gay social engagement including number of friends who are gay (a few (reference group), some, most or all) and time spent with gay friends (a little (reference group), some, most and all), and sexual practices in the preceding six months including number of sex partners (one (reference group), 2–5, 6–10, 11–20, 21–50 or more than 50), and unprotected anal intercourse, separately for regular and casual partners (UAIR and UAIC respectively, both coded as no partners, no anal sex, all sex with condoms or some sex without condoms).

We also used the RDS sample from the CONNECT-I study and RDS partition analysis to produce population estimates of the indicators considered in this analysis. Table 1 presents crude proportions and the asymptotically unbiased prevalence. The RDSII estimator was used to derive sampling weights for each variable for further calculation of asymptotically unbiased estimates. Bootstrapping with 1,000 replicates was used to calculate the population prevalence confidence intervals. Although these estimates are unlikely to be free from bias, they are more likely to be less biased than estimates from the selected samples due to the use of weighting for the probability of selection of recruits. [10] We compared the sample proportions across the studies that used different recruitment methods, specifically convenience time-location sampling, RDS and convenience online sampling, and also compared the sample estimates with the population level estimates produced by RDS partition analysis. The only other data source that previously produced population estimates of interest was the telephone-based survey used by the Australian Study of Health and Relationships (ASHM). [19], [20] Because it was conducted almost a decade prior to the selected studies (in 2003) and had limited number of indicators of interest, it had limited value for our comparison.

**Table 1:** Estimated population prevalence of socio-demographic and behavioural factors among gay men: CONNECT I study (estimates derived using respondent-driven sampling).

Variables	Crude proportion(n)N = 937 1%	Point estimate ofthe populationadjusted proportion (%)	95% bootstrappedconfidence interval for thepopulationadjusted proportion (%)
**Age group (years)**			
<25	15.8% (146)	14.9%	9.2%–21.9%
25–34	37.0% (341)	38.8%	30.7%–46.5%
35–44	25.0% (230)	26.3%	19.7%–31.7%
45–54	15.4% (142)	13.7%	8.9%–20.2%
55+	6.8% (63)	6.4%	3.2%–10.9%
**Ethnic background**			
Anglo-Australian	61.6% (570)	62.9%	56.7%–68.8%
Other	38.4% (356)	37.1%	31.2%–43.3%
**Education**			
Up to 3 years HS	6.7% (62)	5.7%	3.0%–8.0%
Year 12/HSC/TEE	16.7% (155)	20.5%	15.7%–25.4%
Tertiary diploma	24.2% (224)	24.9%	20.5%–30.7%
University level	52.4% (486)	48.9%	42.5%–55.0%
**Ever tested for HIV**			
No	9.4% (86)	10.7%	6.2%–16.6%
Yes	90.6% (832)	89.3%	83.4%–93.8%
**HIV serostatus**			
positive	**11.7%** (110)	7.7%	4.1%–11.3%
negative	75.5% (707)	78.0%	72.3%–83.8%
Don’t know/nor sure	12.8% (120)	14.3%	9.5%–19.9%
**Sexual identity**			
Gay/homosexual	94.2% (855)	96.0%	94.0%–98.1%
Bisexual	3.0% (27)	1.9%	0.5%–3.4%
Heterosexual	0.1% (1)	0.2%	0%–0.4%
Other	2.8% (25)	2.0%	0.5%–3.7%
**Gay friends**			
A few	0.6% (5)	0.2%	0%–0.8%
Some	**17.3%** (158)	26.2%	20.7%–33.0%
Most	31.7% (290)	34.5%	29.2%–40.4%
All	**50.6%** (463)	39.1%	31.6%–45.3%
**Time spent with** **gay friends**			
A little	**0.5%** (5)	0.0%	–
Some	**13.7%** (126)	20.2%	15.0%–27.5%
Most	39.1% (359)	39.1%	33.0%–44.8%
All	46.6% (428)	40.7%	32.7%–47.7%
**No sex partners** **(last 6 months)**			
One	24.7% (217)	31.5%	24.7%–38.0%
2–5	7.3% (64)	8.3%	5.2%–12.0%
6–10	28.6% (252)	32.1%	26.6%–38.5%
11–20	15.1% (133)	12.5%	8.8%–17.1%
21–50	**21.0%** (185)	13.6%	9.4%–17.4%
>50	3.3% (29)	2.0%	0.7%–3.7%
**Anal sex with** **regular partners**			
No partners	45.8% (360)	41.2%	33.1%–47.2%
No anal sex	5.0% (39)	5.1%	2.1%–8.5%
All anal sex with condoms	11.1% (87)	8.4%	5.5%–13.2%
Some anal sex without condoms	**38.2%** (300)	45.3%	38.5%–52.9%
**Anal sex with** **casual partners**			
No partners	30.9% (281)	37.2%	30.5%–44.1%
No anal sex	4.3% (39)	4.6%	2.0%–7.3%
All anal sex with condoms	29.6% (269)	28.2%	22.7%–33.8%
Some anal sex without condoms	35.3% (321)	30.1%	24.5%–36.3%

1 Denominators for individual variables may differ from the grand total due to some missing responses.

Note; Highlighted in bold are variable categories where the crude estimates fall outside the asymptotically unbiased estimates of the 95% bootstrapped confidence intervals.

As the CONNECT–I study was conducted in NSW, Victoria and Western Australia, and population estimates of interest could be obtained for only these three jurisdictions, we limited the samples from all studies to only those participants who reported living in these three states.

We used Pearson’s χ2 test for independence and logistic regression with Type I error of 5% to compare the proportions. All analyses were executed in STATA 12.0 (StataCorp, College Station, TX, USA).

## Results

The crude and estimated population proportions of socio-demographic and behavioural characteristics of gay men in three major Australian states of New South Wales, Victoria and Western Australia are presented in Table 1. The majority of Australian GMSM was estimated to identify as gay or homosexual, and to report high levels of gay social engagement. Estimated HIV testing rates were high, with 89.3% (83.4–93.8%) having ever been tested. Population levels of unprotected anal intercourse were estimated to be at 45.3% (38.5%–52.9%) with regular partners and 30.1% (24.5%–36.3%) with casual partners. The comparison of crude and population-adjusted proportions shows that crude proportions for most variable categories fall within the confidence limits of the asymptotically unbiased estimates of the population-adjusted proportions, indicating little relative bias.

The distributions of the same socio-demographic and behavioural factors in the samples recruited using different recruitment approaches are presented in Table 2. GCPS includes 10,842 men, who were recruited using convenience time-location sampling. CONNECT-I recruited 937 men using RDS, CONNECT-II recruited 667 men online through a single website and PASH recruited 2,306 GMSM online through various websites. GCPS was a behavioural surveillance sample and was used as a reference group in comparisons of the socio-demographic and behavioural characteristics across the samples. CONNECT-I was generally similar to the GCPS with respect to age (except that it had a significantly higher proportion of men aged 25–34 as compared to the rest of the sample). Both online-recruited samples had more age differences compared to the GCPS than CONNECT-I. The proportion of Anglo-Australian men was significantly lower in CONNECT-I and PASH than in the GCPS. As to level of education and having ever being tested for HIV, men in the CONNECT-I were largely similar to those in the GCPS sample; the online samples were less similar. CONECT-I and PASH were similar to the GCPS with respect to HIV serostatus. Significant differences were observed across the samples with respect to sexual identity, particularly the online samples recruited significantly higher proportions of bisexual men and, respectively, lower proportions of men who identified themselves as gay or homosexual. Significant differences were observed across all studies with respect to gay social engagement (number of gay friends and time spent with gay friends). Participants in both CONNECT studies were different from men in the GCPS in terms of the number of sex partners, while men in PASH were similar to the GCPS participants in this regard. All studies found different prevalence rates of UAIC and UAIR.

**Table 2:** Comparison of sample proportions of socio-demographic and behavioural factors among gay men recruited using different sampling methods.

Study name(Sampling method)	GCPS(Time-location)	CONNECT I(Respondent-driven)		CONNECT II(Internet-based, asingle website)		PASH(Internet-based, multiplewebsites)	
Variables	Samplepropor tion	Sample propor tion	Chi2 testp-value for thecomparisonwith GCPSsample	Samplepropor tion	Chi2 test p-value forthe comparison withGCPS sample	Samplepropor tion	Chi2 test p-value for thecomparison withGCPS sample
	N = 10,842	N = 937		N = 667		N = 2,306	
**Age group (years)**							
<25	15.1	15.8	0.557	12.33	0.065	21.3	**0.000**
25–34	32.6	37.0	**0.007**	21.11	**0.000**	30.1	**0.043**
35–44	27.1	25.0	0.161	24.66	0.197	27.1	0.978
45–54	16.8	15.4	0.269	26.52	**0.000**	15.1	0.078
55+	8.4	6.8	0.101	15.37	**0.000**	6.5	**0.008**
**Ethnic background**							
Anglo-Australian	65.9	61.6	**0.008**	69.4	0.074	62.9	**0.007**
Other	34.2	38.4	**0.008**	30.6	0.074	37.1	**0.007**
**Education**							
Up to 3 years HS	7.3	6.7	0.515	10.2	**0.008**	12.1	**0.000**
Year 12/HSC/TEE	18.0	16.7	0.326	15.0	0.061	13.9	**0.000**
Tertiary diploma	20.6	24.2	**0.011**	26.7	**0.000**	20.4	0.831
University level	54.1	52.4	0.326	48.1	**0.005**	53.6	0.688
**Ever tested for HIV**							
No	10.6	9.4	0.233	20.9	**0.000**	15.2	**0.000**
Yes	89.4	90.6	0.233	79.1	**0.000**	84.8	**0.000**
**HIV serostatus**							
positive	10.3	11.7	0.17	8.1	0.067	9.4	0.193
negative	75.7	75.5	0.848	60.0	**0.000**	75.4	0.711
Don’t know/nor sure	14.0	12.8	0.329	31.9	**0.000**	15.2	0.114
**Sexual identity**							
Gay/homosexual	88.5	94.2	**0.000**	69.7	**0.000**	87.4	0.138
Bisexual	6.3	3.0	**0.000**	27.7	**0.000**	10.3	**0.000**
Heterosexual	2.8	0.1	**0.001**	0.5	**0.004**	0.4	**0.000**
Other	2.4	2.8	0.507	2.1	0.649	2.0	0.228
**Gay friends**							
A few	1.4	0.6	**0.035**	12.1	**0.000**	4.9	**0.000**
Some	16.6	17.3	0.603	34.7	**0.000**	35.6	**0.000**
Most	36.7	31.7	**0.003**	31.1	**0.008**	27.9	**0.000**
All	45.4	50.6	**0.002**	22.1	**0.000**	31.6	**0.000**
**Time spent with** **gay friends**							
A little	1.7	0.5	**0.012**	8.0	**0.000**	7.9	**0.000**
Some	17.1	13.7	**0.008**	36.3	**0.000**	31.6	**0.000**
Most	40.8	39.1	0.318	34.7	**0.004**	39.6	0.269
All	40.4	46.6	**0.000**	21.0	**0.000**	21.0	**0.000**
**No sex partners** **(last 6 months)**							
One	13.9	24.7	**0.000**	12.7	0.524	14.3	0.621
2–5	22.2	7.3	**0.000**	7.9	**0.000**	21.7	0.622
6–10	26.2	28.6	0.111	32.0	**0.022**	25.9	0.815
11–20	14.7	15.1	0.708	19.6	**0.015**	14.3	0.677
20–50	19.0	21.0	0.134	22.5	0.118	20.5	0.095
>50	4.1	3.3	0.237	5.4	0.269	3.3	0.055
**Anal sex with** **regular partners**							
No partners	42.0	45.8	**0.038**	76.6	**0.000**	44.7	**0.019**
No anal sex	6.4	5.0	0.108	2.4	**0.000**	7.2	0.189
All anal sex with condoms	17.5	11.1	**0.000**	5.9	**0.000**	10.5	**0.000**
Some anal sexwithout condoms	34.1	38.2	**0.02**	15.1	**0.000**	37.6	**0.001**
**Anal sex with** **casual partners**							
No partners	38.4	30.9	**0.000**	58.2	**0.000**	31.1	**0.000**
No anal sex	12.9	4.3	**0.000**	5.4	**0.000**	11.1	**0.022**
All anal sex with condoms	28.3	29.6	0.403	15.1	**0.000**	29.2	0.372
Some anal sexwithout condoms	20.4	35.3	**0.000**	21.3	0.593	28.6	**0.000**

We then compared the sample proportions for the same set of variables obtained from each of the four studies with the estimated population proportions (see Table 3). In the GCPS, the distributions of variables measuring age, ethnic background, education, ever being tested for HIV and time spent with gay friends fell within confidence limits estimated for population proportions. The proportion of HIV positive men was overestimated as was the proportion of men identifying themselves as gay/homosexual and heterosexual. Importantly, GCPS overestimated the proportion of men who had 2–5 and more than 20 partners in preceding six months, the proportion of men who always used condoms in anal sex with regular partners and the proportion of men who did not have anal sex with causal partners. CONNECT-I sample proportions were the closest to the population estimates, with only a few differences, most important of which were overestimated proportion of men with 20–50 partners within six months and underestimated proportion of men engaging in UAIR. CONNECT-II sample had significant differences from the population estimates on all variables. PASH fell outside of confidence limits for population estimates of men in the 25 to 34 age group, education, most categories of variables describing gay social engagement and the number of sex partners. Regarding two key indicators of anal sex, all studies underestimated the proportion of men who had UAIR within a six month period and the GCPS samples underestimated the proportion of men having UAIC within a six month period.

**Table 3:** Comparison of the sample and population estimates of prevalence of socio-demographic and behavioural factors among gay men.

Study name(Samplingmethod)		GCPS(Time-location)		CONNECT I(Respondent-driven)		CONNECT II(Internet-based,a single website)		PASH(Internet-based,multiple websites)	
Variables	95% CI for thepopulation estimate	Sampleproportion	Compared withPopulationestimate	Samplepropor tion	Compared withpopulationestimate	Sampleproportion	Compared withpopulationestimate	Samplepropor tion	Comparedwith populationestimate
		N = 10,842		N = 937		N = 667		N = 2,306	
**Age group** **(years)**									
<25	9.2%–21.9%	15.1%		15.8%		12.33%		21.3%	
25–34	30.7%–46.5%	32.6%		37.0%		21.11%	↓	30.1%	↓
35–44	19.7%–31.7%	27.1%		25.0%		24.66%		27.1%	
45–54	8.9%–20.2%	16.8%		15.4%		26.52%	↑	15.1%	
55+	3.2%–10.9%	8.4%		6.8%		15.37%	↑	6.5%	
**Ethnic** **background**									
Anglo-Australian	56.7%–68.8%	65.9%		61.6%		69.4%	↑	62.9%	
Other	31.2%–43.3%	34.2%		38.4%		30.6%	↓	37.1%	
**Education**									
Up to 3 years HS	3.0%–8.0%	7.3%		6.7%		10.2%	↑	12.1%	↑
Year 12/HSC/TEE	15.7%–25.4%	18.0%		16.7%		15.0%	↓	13.9%	↓
Tertiary diploma	20.5%–30.7%	20.6%		24.2%		26.7%		20.4%	↓
University level	42.5%–55.0%	54.1%		52.4%		48.1%		53.6%	
**Ever tested for** **HIV**									
No	6.2%–16.6%	10.6%		9.4%		20.9%	↑	15.2%	
Yes	83.4%–93.8%	89.4%		90.6%		79.1%	↓	84.8%	
**HIV serostatus**									
positive	4.1%–11.3%	10.3%		11.7	↑	8.1%		9.4%	
negative	72.3%–83.8%	75.7%		75.5%		60.0%	↓	75.4	
DK/nor sure	9.5%–19.9%	14.0%		12.8%		31.9%	↑	15.2	
**Sexual identity**									
Gay/homosexual	94.0%–98.1%	88.5%	↑	94.2%		69.7%	↓	87.4	↓
Bisexual	0.5%–3.4%	6.3%		3.0%		27.7%	↑	10.3	↑
Heterosexual	0%–0.4%	2.8%	↑	0.1%		0.5%	↑	0.4	
Other	0.5%–3.7%	2.4%	↓	2.8%		2.1%		2.0	
**Gay friends**									
A few	0%–0.8%	1.4%	↑	0.6%		12.1%	↑	4.9	↑
Some	20.7%–33.0%	16.6%	↓	17.3%	↓	34.7%	↑	35.6	↑
Most	29.2%–40.4%	36.7%		31.7%		31.1%		27.9	↓
All	31.6%–45.3%	45.4%	↑	50.6%	↑	22.1%	↓	31.6	
**Time spent** **with gay friends**									
A little	–	1.7%		0.5%		8.0%		7.9	
Some	15.0%–27.5%	17.1%		13.7%	↓	36.3%	↑	31.6	↑
Most	33.0%–44.8%	40.8%		39.1%		34.7%		39.6	
All	32.7%–47.7%	40.4%		46.6%		21.0%	↓	21.0	↓
**No sex partners** **(last 6 months)**									
One	24.7%–38.0%	13.9%	↓	24.7%		12.7%	↓	14.3	↓
2–5	5.2%–12.0%	22.2%	↑	7.3%		7.9%		21.7	↑
6–10	26.6%–38.5%	26.2%		28.6%		32.0%		25.9	↓
11–20	8.8%–17.1%	14.7%		15.1%		19.6%	↑	14.3	
20–50	9.4%–17.4%	19.0%	↑	21.0%	↑	22.5%	↑	20.5	↑
>50	0.7%–3.7%	4.1%	↑	3.3%		5.4%	↑	3.3	
**Anal sex with** **regular partners**									
No partners	33.1%–47.2%	42.0%		45.8		76.6%	↑	44.7	
No anal sex	2.1%–8.5%	6.4%		5.0		2.4%		7.2	
All anal sexwith condoms	5.5%–13.2%	17.5%	↑	11.1		5.9%		10.5	
Some anal sexwithout condoms	38.5%–52.9%	34.1%	↓	38.2	↓	15.1%	↓	37.6	↓
**Anal sex with** **casual partners**									
No partners	30.5%–44.1%	38.4		30.9		58.2	↑	31.1	
No anal sex	2.0%–7.3%	12.9	↑	4.3		5.4		11.1	↑
All anal sexwith condoms	22.7%–33.8%	28.3		29.6		15.1	↓	29.2	
Some anal sexwithout condoms	24.5%–36.3%	20.4	↓	35.3		21.3		28.6	

↑significantly higher than the population estimate (p-value<0.05).

↓significantly lower than the population estimate (p-value<0.05).

A secondary analysis was conducted using the GCPS and PASH samples, based on the observation that these samples tended to differ from the population estimates in the opposite direction from one another. We carried out simulations using a composite of both samples, with different recruitment ratios of participants recruited in physical venues versus online (1∶1, 1∶2, 1∶3, 1∶4, 1∶5), in order to identify which sample composition was most consistent with population estimates (data not presented; available on request). These simulations demonstrated that as the sample ratio reached 1∶5, the observed sample characteristics became more consistent with the population estimates, particularly with regards to UAIC. Regardless of the recruitment ratio, we still observed differences between the composite sample and the population estimates with regards to UAIR, number of sexual partners, and sexual identification.

## Discussion

In the current study, we identified differences in the social and behavioural profile of adult GMSM based on how they were recruited, using a number of sampling approaches commonly employed in behavioural surveillance research. Additionally, we presented the population estimates for a number of socio-demographic and behavioural characteristics of GMSM in three major Australia states. Three primary sampling methodologies were utilized: time-location, RDS, and online recruitment. For the latter, two separate samples were recruited via either a single or multiple websites. All samples were recruited from the same source population and collected the same information, which allowed assessing the scope of comparability between the samples recruited using different recruitment methods. Each sample was compared against RDS-derived population estimates with regards to demographic, social and behavioural indicators commonly measured in behavioural surveillance research amongst GMSM.

Men recruited through time-location sampling (GCPS) shared a similar socio-demographic profile as the population estimated by RDS, and no differences were noted in HIV testing or serostatus. These men appeared more connected to the gay community, spending a greater amount of time with a larger number of gay friends then the overall population, which is unsurprising given sample ascertainment. These men also reported a greater number of sexual partners, although they engaged in less unprotected anal intercourse (with either regular or casual partners). Men recruited through RDS were the most consistent with the population estimates, with no socio-demographic differences noted. The RDS sample slightly overestimated the proportion of HIV-positive men, and also appeared to recruit men with an overall higher number of sexual partners. Few behavioural differences were noted, aside from the RDS sample underestimating the proportion of men engaging in UAIR.

GMSM who were recruited through a single-website survey differed considerably from the population estimates. This sample contained a higher proportion of men aged over 45, was less ethnically diverse, and included a greater proportion of men with high-school only education. These men were less likely to have ever undergone HIV testing, and were more likely to be unaware of their HIV status. This method produced a sample of men who appeared to be less connected to the gay community, and who were more likely to report no sexual contact with either a causal or regular partner in the past six months. In comparison, the online sample recruited through multiple websites was more consistent with population estimates. Overall, few socio-demographic differences were noted, although this sample contained more variation in sexual identification. These men tended towards spending less time with gay friends than the overall population, and had fewer gay friends. These findings are consistent with the profiles described in two previous Australian studies the Private Lives-2 [21] and e-Male [22], [23], which both reported similar patterns of socio-demographic characteristics, sexual identification and HIV testing history. Few behavioural differences were noted in the PASH sample compared to population estimates, although this sample underestimated the proportion of UAIR as well as the proportion of men who did not engage in any anal intercourse with a casual partner.

The socio-demographic profile of GMSM appeared relatively stable across different sampling methods, with greater differences observed amongst the sample recruited through a single website. This approach seems vulnerable to a self-selection bias, given its reliance on recruiting men who have subscribed to a specific website. When men were recruited through a range of websites, the resulting sample was more consistent with the overall population estimates. The social characteristics that varied the most between samples related to gay social engagement patterns. Unsurprisingly, men recruited through time-location sampling appeared to have greater connections to other gay men, likely driven by the use of established gay social venues as recruitment sites and the explicit focus on recruiting community-attached men in the study.

Of particular interest was the stability of key behavioural indicators, such as sexual practices, which are one of the primary outputs of behavioural surveillance amongst GMSM. Each of the separate recruitment methods produced samples that differed from the RDS-derived population estimates with regards to key behavioural indicators such as unprotected anal intercourse with either casual or regular partners. Data acquired through time-location sampling underestimated the rates of both UAIR and UAIC, while RDS and online sampling both generated samples that underestimated UAIR. Given that time-location sampling remains the gold-standard recruitment approach for GMSM behavioural research, the finding that a sample recruited using this methodology underestimated the prevalence of sexual practices strongly related to the transmission of HIV is an important one, particularly in the context of reports of increased rates of UAIC and HIV diagnoses. [24] The lower rates of UAIC in the GCPS sample may reflect the reality that GMSM who utilize the venues that serve as recruitment sites are more likely to be exposed to health-promotion messages than men with fewer connections to the gay community, such as those accessed through online recruitment. In addition, these community-attached men are also more likely to be the target audience for many HIV prevention strategies, such as those emphasizing the risk posed by UAIC in the transmission of HIV.

Of the four recruitment approaches, RDS produced the sample with proportions closest to the RDS-derived population estimates. However, several other factors require consideration when comparing sampling methodologies. Each recruitment approach differed in the level of input required, as well as the value or utility of the data it provided. Time-location sampling, as used in the GPCS, is currently a gold-standard method for recruitment GMSM for behavioural surveillance studies, and is capable of producing samples representative of the overall sampling frame as long as the selection of venues is adequate. Additionally, the GCPS in Australia allows for measuring trends over time, with consistent data collection protocols established from 1996. However, this need for consistency limits the flexibility of the content in the GPCS although there has been some scope for collecting one-off data about specific issues. [25] Further, the use of the same venues over time makes the GCPS sensitive to changes in venue clientele. The growing prominence of online social networks as a way to locate sexual partners may limit the potential for the GCPS to capture GMSM who have shifted away from physical venues, particularly young gay men who are underrepresented in typical GCPS samples. [26] The funding and staffing demands of the GCPS are slightly greater than online data collection, although the study’s profile in the community limits the amount of advertising required.

Online recruitment offers a clear alternative to time-location sampling, particularly given the prominence of the Internet in gay men’s social and sexual networks. [27] It also offers the potential of recruiting a broader sample of GMSM, by including men who are not accessed via traditional location-based recruitment which focus on community-attached gay men. Both the Connect-II and PASH studies had lower costs and logistic demands relative to the GCPS, although advertising requirements were greater. The importance of selecting the websites through which men are recruited appeared critical; the Connect-II study was based on men accessed through a single website, and this sample was perhaps the most divergent from population estimates. The PASH study recruited men through a range of websites and also differed from population estimates with regards to both social and behavioural factors. Intriguingly, the direction of these differences was often in the opposite direction as the differences between the GCPS and the population estimates. Based on our secondary analysis, we observed that combining both venue-based and online recruitment generated a sample with characteristics more consistent to the population estimates than any of the individual recruitment methods. This suggests that online recruitment and time-location sampling tap into overlapping but distinct subgroups with important qualitative differences, and combining the two approaches might offer the potential for recruiting a more inclusive and representative sample of GMSM. Similarly, Guo et al. reported different behavioural and demographic profile amongst Chinese MSM based on sampling methodology, and encouraged the careful selection of multiple recruitment approaches in improving the representativeness of MSM samples [9].

Finally, despite the consistency between population estimates and the group recruited via RDS, this methodology is perhaps too complex and logistically demanding to be easily incorporated into routine behavioural surveillance among GMSM in Australia. This methodology may be more appropriate for investigation of population issues, as well as exploring specific empirical questions rather than ongoing surveillance. It has some value in offering the potential to produce population estimates, as well as utility as a reference for evaluating the reliability of other sampling approaches. Although this methodology can be considered as a potentially superior form of convenience sampling, the results produced by it are still prone to some residual bias and should be interpreted with caution [10].

The current data clearly indicate the potential for different recruitment approaches to produce samples of GMSM with differing social and behavioural profiles. To our knowledge, this is the first study to evaluate the statistical significance of the differences between the samples recruited from the same source population using differing recruitment approaches. The findings suggest the need for careful consideration of the changing nature of social and sexual networks, and the influence this shift has had on data derived from traditional venue-based recruitment methodologies. The current data emphasize the importance of a clear understanding of the relative strengths of each recruitment approach, and the need for a clearly articulated rationale for the selection of a particular method. Rather than a methodological limitation, this highlights an important opportunity for accessing a broader, more representative sample of GSMM by combining traditional time-location sampling with online recruitment. Further investigation of this is necessary, in order to ascertain the most effective and reliable way of gathering the data necessary for providing an empirically sound basis for health-promotion and intervention efforts.
